# Re-Use of Established Drugs for Anti-Metastatic Indications

**DOI:** 10.3390/cells5010002

**Published:** 2016-01-12

**Authors:** Frank Entschladen, Dane A. Thyssen, David W. Drell

**Affiliations:** 1 MetaVì Labs Inc., 16238 Ranch Road 620 North, Suite F-347, Austin, TX 78717, USA; athyssen@metavilabs.com (D.A.T.), ddrell@metavilabs.com (D.W.D.); 2 Faculty of Health—School of Medicine, Witten/Herdecke University, Alfred-Herrhausen-Straße 50, 58448 Witten, Germany

**Keywords:** cancer, metastasis, cell migration, drug screening

## Abstract

Most patients that die from cancer do not die due to the primary tumor but due to the development of metastases. However, there is currently still no drug on the market that specifically addresses and inhibits metastasis formation. This lack was, in the past, largely due to the lack of appropriate screening models, but recent developments have established such models and have provided evidence that tumor cell migration works as a surrogate for metastasis formation. Herein we deliver on several examples a rationale for not only testing novel cancer drugs by use of these screening assays, but also reconsider established drugs even of other fields of indication.

## 1. New Test Methods for Established Drugs Can Reveal Additional Indications

The development process of a new drug from target identification to approval is time-consuming and expensive in any type of medical indication. Therefore, when a drug, once approved, turns out to be well-tolerated and effective, it is mandatory to further explore the profile of efficacy and side effects. On the one hand, possible risks of unwanted side effects have to be monitored in order to protect the patients. On the other hand, there may be some beneficial effects aside from the primary indication, which can only be detected under certain circumstances, e.g., in special subgroups of patients, in combination with other drugs or in long-term use. These beneficial effects may lead to a second or extended approval and maximize the profit for both patients and companies. This issue of drug repositioning especially applies to the oncological field, since the success rate for new drugs is particularly low as compared to non-oncological indications [1]. Even established drugs of other fields of medical indications may be identified as being effective in cancer treatment in the future, as will be discussed below on recent examples.

In order not to be dependent on occasional observations, how can such a new application be tested systematically? New assays for drug testing may reveal further forms of action not only for new drugs, but also for old ones, and may open new fields of applications. The European Medicines Agency (EMA) has claimed new test methods for drug development: “Until non-clinical models with good predictive properties have been defined…the absence of such models is considered to constitute the greatest hurdle for efficient drug development within the foreseeable future.” [2]. This applies not only to new drugs, but, in light of drug repurposing, even old drugs should by tested by means of such new models. These new models comprise tumor cell functions that have not been tested before, e.g., angiogenesis and migration [3], but may also apply to the elaboration of conventional cytotoxicity tests, e.g., by the use of spheroid models instead of classic cell culture. However, herein we focus on the example of anti-metastatic drugs and on tumor cell migration as its valid surrogate. Metastasis formation is the most pernicious event in the course of a cancer disease and is decisive for the patients’ survival probability. Although over 90% of those patients that die from cancer die due to the development of metastases [4], there is still no anti-metastatic drug on the market. The establishment of tumor cell migration assays facilitates the detection of such an anti-metastatic effect. In this regard Patricia Steeg has rendered: “…drug development continues to rely heavily on short-term reductions in the size of primary tumors. These data suggest that compounds validated in this manner may not work on metastatic disease, and that compounds with antimetastatic efficacy may not be validated in tests based on reduction of primary tumor size.” [5]. In this line of argumentation, Mike Fernandes and his colleagues further ask if size-based response criteria might be an anachronism [6]. Alexandra Zimmer and Patricia Steeg have nicely summarized data on metastasis prevention of existing cancer therapeutics including receptor and non-receptor kinase inhibitors [7]. 

## 2. Examples of Drug Repurposing in Oncology

To date, the by far most striking discovery in the context of drug repurposing in oncology is the anti-metastatic function of beta-blockers [8]. Stimulated by results provided by *in vitro* drug testing systems [9,10,11], and confirmed by mice experiments [12], several retrospective patient studies have delivered proof for an anti-metastatic effect in various types of solid tumors, *i.e.*, breast, ovarian, prostate, colorectal, lung, liver, melanoma [13,14,15,16,17,18,19,20,21,22,23,24,25,26,27,28,29]. The *in vitro* drug testing systems that initially led to this development were migration analyses, which are currently not a standard drug screening method in oncology, but may constitute such a new model as requested by the EMA. The function of beta-blockers has been identified by a three-dimensional, collagen-based migration model of single cells [30], but several other tumor cell migration assays have been established that may turn out to be qualified as novel drug screening methods [31,32,33].

The benefits of drug repurposing in oncology have been recently discussed in detail by Pan Pantziarka and his colleagues, providing a list of six generic drug candidates to be tested for oncology application in clinical trials in the ReDo project, namely Mebendazole, Nitroglycerin, Cimetidine, Clarithromycin, Diclofenac and Itraconazole [34]. In addition to these advanced projects, there are several further candidates for such a possible use or re-use of established drugs in oncology. A recent example is the GABA-B receptor agonist baclofen, which was initially approved for the treatment of epilepsia. Current research on the role of GABA in cancer provided conflicting results: some reports support the view that the GABA-B receptor may have a tumor-promoting effect in prostate and breast cancer [35,36], whereas Ortega’s review suggests an inhibitory effect on tumor metastasis in various types of cancer [37]. Neman and colleagues provided the most striking evidence on human tissue samples of breast cancer that the GABAergic system is involved breast-to-brain metastasis formation [38], showing that the GABAergic system is a valid target and existing drugs on the involved receptors may be considered for an oncological application. Another very interesting example is thalidomide. This drug is supposed to have immunomodulatory effects that inhibit angiogenesis in myeloma [39], whereas it has previously been approved as a sedative but sadly turned out to have serious teratogenic side effects [40]. Nevertheless, these new findings on angiogenesis inhibition stimulated the development of novel thalidomide analogues as immunomodulatory and anti-angiogenic drugs [41].

Further neurotransmitters with a proven function in breast cancer cell migration, in addition to the aforementioned adrenergic and GABAergic axes, are dopamine and substance P acting via the D2 and neurokinin-1 receptors, respectively [10]. These effects are inhibited by the respective drugs metoclopramide for the D2 receptor and L-733,060 for the neurokinin-1 receptor. Furthermore, engagement of the inhibitory cannabinoid receptors by its physiological agonist anandamide reduced tumor cell migration of colon cancer cells as well [42]. Therefore, there are several examples of neurotransmitter function in tumor cell migration, and one might speculate that further clinically established agonists or antagonists for neurotransmitters, as listed in Table 1, can have a clinical effect in cancer progression as well. We have found some effects of neurotransmitters on breast cancer cell migration [9], and therefore suggest (re-)evaluating these drugs by *in vitro* test systems and retrospective patients studies in parallel for a potential role in cancer.

**Table 1:** Neurotransmitter agonists and antagonists.

*Proven Effect on Cancer Cells*		
Substance	Current indication	Receptor
Propranolol	hypertension	β1/β2-adrenoceptor-antagonist
Baclofen	epilepsia	GABA-B agonist
Metoclopramide	nausea	dopamine D2 antagonist
L-733,060	anxiety, depression	neurokinin-1 antagonist
***Further Drugs to be Evaluated***		
Substance	Current indication	Receptor
Iosartan	hypertension	angiotensin AT1 antagonist
Ipratropium	asthma	anticholinergic
Montelukast	asthma	leukotriene D4 antagonist
Ioratadine	allergies	histamine H1 antagonist
Famotidine	ulcers	histamine H2 antagonist
Olanzapine	schizophrenia	dopamine D1/D2/5-HT2
Risperidone	psychosis	dopamine D2/5-HT2A
Sumatriptan	migrane	serotonin 5-HT1 agonist
Fentanyl	pain	opioid agonist

## 3. Selection of Candidates by Their Action in Signal Transduction

**Figure 1:**
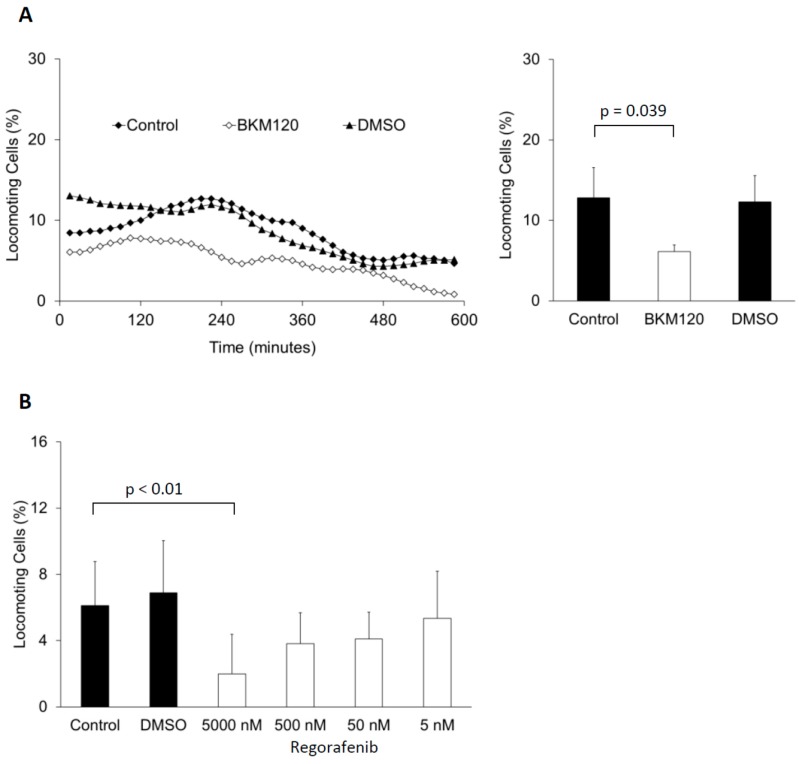
Cell migration was performed using the human estrogen receptor–positive, luminal-like breast carcinoma cells MCF-7 with regard to BKM120 (*n* = 3; **A**); and the human epitheloid pancreatic cancer cell line PANC-1 with regard to Regorafenib (*n* = 7; **B**). Due to limited availability of material, only three experiments have been performed with one concentration for BKM 120. Both cell lines were obtained from the Cell Lines Service GmbH (Eppelheim, Germany). DMSO concentrations correspond to the highest concentration used to dissolve the substances. In (**A**), the left graph shows the time course of the migratory activity, the right graph displays the mean activities and standard deviations after four hours; In (**B**), the columns show the mean activities and standard deviations of the entire observation time of 10 h.

The aforementioned examples have provided evidence that old drugs can successfully be reconsidered for an oncological indication. By means of tumor cell migration assays as novel drug screening models, new or existing drugs can be tested for anti-metastatic effects, and we have exemplarily investigated two existing oncology drugs that were, from their mode of action, likely candidates. These are Bayer’s multi-receptor tyrosine kinase (RTK) inhibitor Regorafenib (Figure 1A) and Novartis’ phosphatidyl-inositol-3 kinase (PI3K) inhibitor BKM120 (Buparlisib; Figure 1B). We had selected these drugs since they are both small molecule kinase inhibitors with a known involvement in the signal transduction that regulates tumor cell migration [43]. RTKs are the receptors for growth factors and other members of the cytokine super-family, which are known regulators of migratory activity. With regard to cancer, the epithelial growth factor receptor (EGFR) has probably received the most interest, especially the family member HER2 (also known as erbB2). HER2-positive tumors tend to be more aggressive than other types of breast tumors [44]. The PI3K is a downstream element in signal transduction, directly activated by RTKs [43]. The PI3K generates the two second messengers, diacylglycerol and inositol-trisphosphate, by the enzymatic breakdown of phosphatidyl-inositol-bisphosphate. Inositol-trisphosphate induces a release of calcium from intracellular stores, and diacylglcerol is an activator of the protein kinase C. Both second messengers, in concert, regulate actin polymerization, actin-binding molecules, and non-muscle myosin II activity [43], whereas the sole activation of the protein kinase C is a strong inducer of tumor cell migration [45].

We have used the same cell migration assay that was previously used for the discovery of the anti-migratory effect of beta-blockers [9,10,11]. This assay is a single-cell migration assay, with the cells being embedded into a three-dimensional collagen matrix close to physiological conditions. As shown in Figure 1, both of the investigated substances significantly inhibited tumor cell migration at concentrations that are relevant for patients’ treatment (the concentrations were calculated on the basis of the clinically applied doses). Thus, these results would support the claim of having an anti-metastatic effect for these drugs.

For the analysis of the migratory activity, the 10 h observation period is subdivided into 15 min intervals. The term “locomoting cell” is defined as a cell which moved within a particular observation period (each period is marked in Figure 1A (left) by a symbol). The *y*-axis shows the locomoting cells as part of the entire observed population (in percent) for each of these intervals. For the column diagrams (A right and B), the average value of the locomoting cells of the observation intervals has been generated independently for each single experiment. The columns display the subsequently generated mean values and standard deviations of all experiments that have been performed. Please note, that the standard deviation appears to be higher in Figure 1B as compared to Figure 1A (right), since the overall activity of PANC-1 cells is lower than that of MCF-7 cells. In addition, in Figure 1B the entire observation period of 10 h has been chosen to generate the over-time average, which caused a higher inter-experimental deviation than the 4 h period of Figure 1A (right). For details on the analysis of migration parameters, please see Niggemann *et al.* [46].

## 4. Conclusions

We have shown herein, on the example of tumor cell migration, that in addition to the search for new drugs by means of conventional preclinical test systems, the establishment of novel test systems can help not only the development of new drugs but is also valuable for discovering further functions of existing drugs. The advantages of the latter are (i) for the patients for whom established drugs are already clinically characterized and thus have a known profile of safety and action, (ii) for the pharma companies for whom an extension of applications increases the drugs’ value. With regard to anti-metastatic drugs, it has turned out, on the example of beta-blockers, that cell migration is a valid preclinical test system for their identification. In this regard, the knowledge of the molecular signal transduction that regulates tumor cell migration provides a systematic approach for the selection of anti-metastatic drug candidates.
